# Evaluating Postoperative Prognosis: A Single Surgeon's Experience With Total Mesorectal Excision in Middle and Lower Rectal Cancer Cases in Iraq

**DOI:** 10.7759/cureus.62174

**Published:** 2024-06-11

**Authors:** Aqeel S Mahmood, Mohammed Ahmed Abbas, Ammar Gany Yassin, Haider A Ahmed, Samer Sabri, Ahmed A Shakir, Hussein Abbas, Mustafa Ismail

**Affiliations:** 1 Department of Surgery, College of Medicine, University of Baghdad, Baghdad, IRQ; 2 Department of Biochemistry, College of Medicine, University of Karbala, Karbala, IRQ; 3 Department of Surgery, Iraqi Board of Medical Specializations, Baghdad, IRQ; 4 Department of Surgery, Baghdad Teaching Hospital, Baghdad, IRQ

**Keywords:** adenocarcinoma, short-term outcomes, neoadjuvant therapy, total mesorectal excision, rectal cancer

## Abstract

Introduction: Surgical intervention for rectal cancer is widely recognized for its potential to significantly impact quality of life, chiefly due to the high probability of permanent colostomy and the associated postoperative complications.

Objective: This study aimed to evaluate the short-term outcomes and morbidity associated with total mesorectal excision for middle and lower rectal cancer within an Iraqi cohort, in a prospective setting.

Methods: This study prospectively collected and analyzed data from 89 patients who underwent a standardized radical rectal resection, with a follow-up period extending to one month post-surgery.

Results: The mean age of patients was 54.4 ± 12.9 years, with a gender distribution of 46 males and 43 females. A total of 33 patients presented with preoperative comorbidities, which heightened the risk of adverse short-term outcomes by a factor of 7.51. The most prevalent comorbidities were hypertension and diabetes mellitus, affecting 22 and 20 patients, respectively. Patients aged 60 years and above were at a 3.97 times greater risk of developing complications. The overall complication rate was 21.35%, with wound infections (9.0%) and cardiovascular events (3.4%) being the most common. Mortality during the follow-up was 1.1%.

Conclusion: The findings indicate that increased age and the presence of comorbidities are significant risk factors for morbidity and mortality post-surgery. Neoadjuvant chemoradiotherapy or radiotherapy was shown to reduce morbidity and mortality rates while improving survival. The morbidity and mortality rates observed in this study concur with existing literature.

## Introduction

Rectal cancer represents a significant public health concern, being one of the predominant malignancies within Western countries, emerged as the third most common cancer around the world, and ranks the fourth most frequent cause of cancer-related death [1]. Its incidence, morbidity, and resource utilization reflect the challenges faced in making an effective management scheme.

It exhibits a pronounced predominance in males and individuals aged 50 and above [2]. The contemporary cornerstone of surgical intervention involves a combination of proctectomy and mesorectal excision, designed to conserve the anal sphincter [3]. Abdominoperineal (AP) resection is selectively employed for cases where the cancer's inferior pole is situated less than 2 cm from the anal sphincter or, alternatively, in specialized instances where the anal canal itself is compromised [4]. Total mesorectal excision (TME), a technique pioneered by Heald in 1982 [5], has become the benchmark surgical strategy for addressing rectal cancer. Prior to the advent of TME, local recurrence rates were alarmingly high, reaching up to 25% [6]. The principal objective of TME encompasses the comprehensive removal of the rectal tumor alongside the pararectal lymph nodes, key sites for initial lymphatic dissemination of tumor cells, while meticulously preserving the adjacent structures, notably the nerve fibers integral to the functioning of the urinary bladder, prostate, and vagina. The intricate pelvic anatomy and the limited space for surgical navigation render TME a particularly challenging procedure [7].

Anastomotic leakage represents a significant complication in sphincter-preserving surgeries for rectal cancer. Various studies reported incidence rates that varied with the advancement of surgical techniques. A 1.3% incidence rate was reported by Shin et al. in their study population [8]. Later on, Yu et al. reported an anastomotic leakage rate of 4.7% after laparoscopic sphincter-preserving surgery [9]. These findings indicate that improvements in surgical practices have enhanced patient outcomes and reduced mortality rates.

The destructive nature of rectal cancer surgery, coupled with the potential for definitive colostomy, incontinence, sexual and urinary dysfunctions, not to mention the profound alterations in body anatomy and overall diminution in quality of life, underscores the gravity of this condition. This study aims to evaluate the short-term outcomes and morbidity associated with TME in patients diagnosed with middle and lower rectal cancer, drawing from a prospective analysis based on the singular experience of a surgeon operating within both governmental and private hospital settings in Iraq.

## Materials and methods

Study design and setting

This investigation was conducted as a prospective cohort study at Baghdad Teaching Hospital/Medical City, alongside various private healthcare facilities in Baghdad. The study period spanned from January 1, 2016, to December 30, 2019.

Study population and eligibility criteria

A total of 89 patients' data were meticulously evaluated within this study. The study cohort inclusion criteria included individuals diagnosed with rectal adenocarcinoma, as confirmed via biopsy, who underwent standardized radical rectal resection in the mentioned place and timeline. A comprehensive assessment revealed that all tumorous growths were located 1 to 10 cm from the dentate line. Exclusion criteria included patients with histopathological diagnoses other than adenocarcinoma, patients who underwent palliative or nonstandardized resections, and patients with incomplete medical records or follow-up data.

Interventions and management

In our study, 65 patients received long-course chemoradiotherapy (LCRT), which consisted of 45-50.4 Gy in 25-28 fractions over 5-6 weeks with concurrent chemotherapy (5-fluorouracil or capecitabine), followed by surgery after a 6-8-week interval. Nine patients underwent short-course radiotherapy (SCRT), involving 25 Gy in five fractions over one week, followed by surgery within 1-2 weeks. The choice between SCRT and LCRT was based on tumor characteristics and clinical guidelines, with SCRT typically chosen for resectable locally advanced rectal cancer and LCRT preferred for cases with a higher likelihood of positive surgical margins [10,11]​. The surgical approach for all curative-intent radical resections of rectal cancer was uniform, focusing on the meticulous mobilization of the rectum and the attainment of clear proximal, lateral, and radial margins. This was contingent upon the level of restorative anastomosis implemented, encompassing anterior resection, low anterior resection, ultra-low anterior resection, and AP resection, all of which were executed via a lower midline incision. Postoperative follow-up was conducted over a span of one month.

All patients received perioperative antibiotic prophylaxis and underwent mechanical bowel preparation to reduce bacterial load. Strict aseptic techniques and sterile environments were maintained during surgery. Intraoperative measures included minimizing tissue trauma and preventing bowel content spillage, with thorough irrigation of the surgical field. Postoperatively, patients were closely monitored, with regular sterile wound care and early ambulation to promote circulation. Adequate nutritional support was also provided to enhance recovery and support the immune system. These comprehensive strategies were essential in minimizing postoperative infections.

Data collection and analysis

Data were collected prospectively and included descriptive statistics to summarize patient demographics, clinical characteristics, treatment details, and short-term postoperative outcomes. Complications were recorded and classified as either local or systemic. The primary outcomes of interest were morbidity and mortality within the one-month postoperative period. Data analysis utilized IBM SPSS Statistics for Windows, Version 25 (Released 2017; IBM Corp., Armonk, New York, United States). The association between comorbidities and postoperative complications was evaluated using the chi-square test or Fisher's exact test, as appropriate. Logistic regression analysis was performed to identify factors associated with an increased risk of postoperative complications. A p-value of less than 0.05 was considered statistically significant.

Ethical considerations

This study was conducted according to the Helsinki Declaration after approval from the Institutional Review Board at Baghdad Teaching Hospital/Medical City (Iraqi Board of Medical Specialization). All the participants were enrolled in the study after individual informed consent. Patient confidentiality was observed during data collection, and coding was only done afterward. The participants in the study were informed and told verbally that they had the complete right to withdraw from the research at any given time point without affecting medical care in their lives. All the interventions and procedures given to the patient in this case were availed by a team of competent medical personnel, where the welfare of the patient was of paramount importance.

## Results

The demographic spread of the study participants ranged from 24 to 81 years, with an average age of 54.4 ± 12.9 years. Males constituted 51.7% of the sample, amounting to 46 individuals. The distribution of cancer location within the cohort revealed 48 (53.9%) patients with middle rectal cancer and 41 (46.1%) patients with lower rectal cancer. It was observed that individuals aged 60 years and above were 3.97 times more likely to experience complications, a finding that was statistically significant (p < 0.05, logistic regression analysis). A noteworthy response to chemoradiotherapy was documented, with 15 (16.85%) patients exhibiting complete regression, 49 (55.06%) demonstrating down-staging (p < 0.05, chi-square test), and 10 (11.24%) showing no response to the treatment (Table 1, data represented as N and %).

**Table 1:** Demographic data and treatment modalities

Baseline Characteristic	Full Sample (n=89)	
	n	%
Gender		
Female	43	48.3
Male	46	51.6
Age		
20-29 Years	5	5.6
30-39 Years	6	6.7
40-49 Years	14	15.7
50-59 Years	29	32.5
60-69 Years	26	29.2
≥70 Years	9	10.1
Tumor Site		
Middle Rectum	48	53.9
Lower Rectum	41	46.1
Pγ Staging		
0	16	17.9
1	44	49.4
2	23	25.8
3	6	6.7
Clinical Staging		
0	2	2.2
1	22	24.7
2	10	11.2
3	55	61.7
Rectal Tumor Grading		
High Grade Dysplasia	1	1.1
Well Differentiated	4	4.4
Moderately Differentiated	79	88.7
Poorly Differentiated	5	5.6
Surgical Procedures		
Anterior Resection	38	42.6
AP-Resection	26	29.2
Low Anterior Resection	18	20.2
Ultra Low Anterior Resection	7	7.8
Neoadjuvant Therapy		
Chemoradiotherapy	74	83.1
Pathological Response to Therapy		
Not Received	15	16.8
Complete Regression	15	16.8
Downstaging	49	55.0
Not Responded	10	11.2
Smokers	26	29.2

The surgical intervention entailed standardized radical rectal resection with TME for all participants. Histopathological examination confirmed adenocarcinoma in all specimens, predominantly of moderate differentiation. The prevalence of comorbid conditions was significant, with hypertension and diabetes mellitus observed in 24.72% (22 patients) and 22.47% (20 patients) of the patients, respectively, followed by cardiac issues in 13.48% (12 patients) (Table 2, data represented as N and %).

**Table 2:** Preoperative comorbidities of the study population HT: Hypertension; DM: diabetes mellitus; COPD: chronic obstructive pulmonary disease

Comorbidities	Number	Percent
Patients with No Comorbidities	56	62.92
HT	22	24.72
DM	20	22.47
Cardiac Comorbidities	12	13.48
Renal Impairment	3	3.37
COPD	2	2.47
Classes Are Not Mutually Exclusive		

The analysis revealed a statistically significant correlation between the presence of comorbidities and the development of complications (p < 0.01, chi-square test). Specifically, 45.5% (20 out of 44) of patients with comorbid conditions encountered complications, in stark contrast to 8.9% (4 out of 45) of those without such conditions.

**Figure 1:**
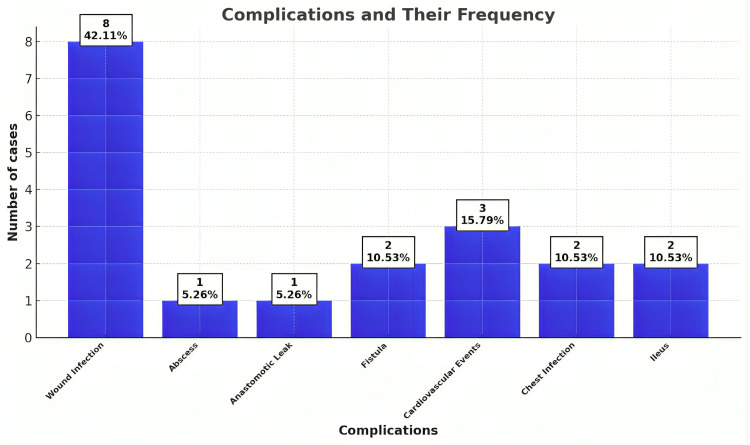
Distribution of the study sample according to complications

Postoperative short-term outcomes indicated a complication rate of 21.35% (19 out of 89 patients), with eight male patients (8.99%) and 11 female patients (12.36%) affected. One patient (1.1%) died during the follow-up period. This patient was a 62-year-old male with a history of hypertension, diabetes mellitus, and cardiac comorbidities. He developed ventricular fibrillation due to ST-elevation myocardial infarction on the first postoperative day, which subsequently led to cardiac arrest. The logistic regression analysis revealed that age and the presence of comorbidities were significant predictors of postoperative complications (p < 0.05). Among the 19 patients who developed complications, wound infection was the most common, affecting eight patients (9.0%), followed by cardiovascular events, ileus, and anastomotic fistula. These findings highlight the varied spectrum of postoperative challenges encountered, emphasizing the need for vigilant clinical management and follow-up (Table 3, data represented as N and %) (Figure 1, data represented as N and %). The difference in the distribution of complications was statistically significant (p < 0.05, Fisher's exact test) (Tables 4-6).

**Table 3:** Morbidity and mortality among the study group

Short-Term Outcome	Number	Percent
Abscess	1	1.1
Anastomotic Leak	1	1.1
Cardiovascular Events	3	3.4
Chest Infection	2	2.2
Fistula	2	2.2
Ileus	2	2.2
Wound Infection	8	9.0
Mortality	1	1.1
No Complications	69	77.52
Total	89	100.0

**Table 4:** Multivariate binary logistic regression for different variables as predictors for developing morbidity/mortality AP: Abdominoperineal; ULR: unilateral lateral rectus recession

Variables	Odd’s ratio	95% CI^*^		P-value
		Lower	Upper	
Age ≥ 60 years	3.97	1.39	11.33	0.010
Comorbidities	7.51	2.38	23.71	<0.001
Female Gender	1.41	0.52	3.84	0.498
Lower Rectum	1.38	0.50	3.78	0.537
Pγ staging	-	-	-	-
Stage I Compared to stage 0	0.91	0.17	4.77	0.913
Stage II Compared to stage 0	1.82	0.44	7.46	0.407
Clinical Staging				
Stage II Compared to stage I	1.12	0.21	6.12	0.892
Stage III Compared to stage I	2.10	0.68	6.49	0.198
Grade				
Well Differentiated Compared to Poorly Differentiated	0.35	0.05	2.29	0.275
Moderately Differentiated Compared to Poorly Differentiated	1.50	0.11	21.31	0.765
Surgery				
Ant. Resection Compared to ULR	2.14	0.23	20.06	0.504
AP Resection Compared to ULR	1.80	0.18	18.05	0.617
Low ant. Resection Compared to ULR	1.20	0.10	13.95	0.884
Neoadjuvant Therapy	0.51	0.15	1.71	0.275
Smoking	1.05	0.35	3.12	0.930
Stage III Pγ Was Excluded As All of Them Had No Complications, Stage 0 Clinical Staging Was Excluded As Only Two Patients Had It. CI*: Confidence Interval.				

**Table 5:** Distribution of the study variables according to improvement in staging and complications

Pathological Staging Compared to Clinical Staging	Complications		Total	P-value*
	No	Yes		
	No.(%)	No.(%)	No.(%)	
Complete Regression	13(86.7)	2(13.3)	15(100)	0.667
Down Staging	38(77.6)	11(22.4)	49(100)	
Not Responded	8(80)	2(20)	10(100)	
Not Received Chemoradiotherapy	10(66.7)	5(33.3)	15(100)	
Total	69(100)	20(100)	89(100)	
*:Fisher’s exact test				

**Table 6:** Distribution of the study variables according to improvement in staging and comorbidities

Pathological Staging Compared to Clinical Staging	Comorbidities		Total	P-value
	No	Yes		
	No.(%)	No.(%)	No.(%)	
Complete Regression	9(60)	6(40)	15(100)	0.614
Downstaging	8(80)	2(20)	10(100)	
Not Responded	31(63.3)	18(36.7)	49(100)	
Not Received Chemoradiotherapy	8(53.3)	7(46.7)	15(100)	
Total	56(62.9)	33(37.1)	89(100)	
Chi-square Test				

## Discussion

In the current study, the most frequently represented age groups were 50-59 and 60-69 years, with a nearly equal distribution of genders. Further analysis indicated that individuals aged ≥ 60 years and females are at an increased risk for morbidity and mortality. These findings are in contrast to those of Purim et al., who examined 40,692 patients with rectal cancer and reported that approximately 65% of patients were aged 65 years or more. The distribution of stage at diagnosis was similar between men and women [12]. Conversely, a study by Kleemann et al. involving 1000 patients with rectal cancer reported no gender-related differences in the oncologic surgical treatment outcomes of patients with rectal carcinoma, although male gender appeared to be a risk factor for increased early postoperative morbidity [13]. This discrepancy might be attributed to the higher prevalence of comorbidities among older age groups.

In our study, 21.3% of the sample developed complications, and only one patient (1.1%) died during the follow-up period. The most common local complications were wound infection and fistula, while systemic complications predominantly included cardiovascular complications and chest infections. Earlier studies, such as Bokey et al., reported complication rates of 25.5% and a mortality rate of 3.6%; respiratory complications were observed in 13.5%, wound infections in 11.1%, and cardiac complications in 5.9% [14]. Alves et al. analyzed 238 patients with rectal cancer and found a morbidity rate of 43% and a mortality rate of 2.5%; systemic complications included 5.5% cardio-respiratory disorders, while local complications included 8% abdominal wall abscess and 7.1% anastomotic leak (AL) [15].

Another large study discussed postoperative outcomes for resection of rectal cancer and reviewed 36,000 patients, of whom over half have undergone radical surgery for rectal cancer. The AL rate was found to be 11%, the rate of pelvic sepsis was 12%, and the postoperative mortality rate was 2%. Besides, wound infections were noted in 7% of the cases. This huge series points out in a clear way the high complexity of rectal cancer surgery and underlines the indispensable, very thorough postoperative care and impeccable surgical technique to reduce as much as possible the number of complications and improve the results for the patient [16].

Infections remained a predominant complication postrectal cancer surgery, with risk factors including colostomy formation, preoperative radiation, and steroid use. Some investigators have even linked postoperative infections to long-term cancer recurrence [17]. Serra-Aracil et al. noted that despite ideal preventive measures, the infection rate remained higher than expected [18], potentially due to unavoidable contamination by fecal material.

Regarding pelvic abscess, one (1.1%) patient developed this complication, which is lower compared to the study by Vermeer et al., who reported a 9.7% incidence of presacral abscess in a cohort of 517 patients with rectal cancer [19]. In addition to the previously mentioned risk factors for infection, drain obstruction could also contribute to abscess formation.

In this study, postoperative fistula formation was observed in two (2.2%) patients, which is lower than the rate reported by Muratore et al., who found a 7.7% incidence among 26 patients undergoing laparoscopic TME [20].

AL occurred in one (1.1%) patient in our study, a rate lower than that reported by Paun et al. in a systematic review and meta-analysis of 84 studies, where 11% of patients developed AL following rectal cancer surgery [16]. The technique of anastomosis construction, whether stapled or handsewn, may influence the prevalence of this complication, with some advocating no significant difference in AL rates between the two methods.

This study has several limitations that should be acknowledged. First, the sample size of 89 patients, while adequate for initial observations, may not be sufficient to draw definitive conclusions applicable to the wider population. Second, the study was conducted at a single institution with the surgeries performed by a single surgeon, which may introduce bias and limit the generalizability of the findings. Third, the follow-up period of one month is relatively short and may not capture long-term complications or outcomes that could arise after the initial postoperative phase. Additionally, data collection relied on the accuracy and completeness of medical records, which can sometimes be subject to errors or omissions. Finally, while the study attempted to control for comorbidities and other variables, there may still be confounding factors that were not accounted for, potentially impacting the results. Future research with larger, multicenter cohorts and extended follow-up periods is necessary to validate these findings and provide a more comprehensive understanding of the short-term and long-term outcomes of TME in rectal cancer patients.

## Conclusions

This study highlights that patients aged 60 and above, and those with comorbidities such as hypertension and diabetes, face higher postoperative risks following TME for rectal cancer. With a complication rate of 21.35%, primarily involving wound infections and cardiovascular events, the findings align with existing literature and emphasize the procedure's complexity. Neoadjuvant therapies were beneficial in reducing morbidity and improving survival. These key findings imply that targeted preoperative interventions could significantly improve patient outcomes. Future research should aim for larger, multicenter studies with extended follow-up to validate these findings and optimize patient care strategies.

## Appendices

**Table 7:** Raw data for the included cohort DM: Diabetes mellitus; HT: hypertension; AP: abdominoperineal

ID	Sex	Age	AgeGroups	Site Tumour	p y Staging	Clinical Staging	P Y minus Clinical	TNM	Grade	Surgery	Neoadjuvant	Previous Surgery	Comorbidities	Smoking	Complications
1	F	57	5	Middle Rectum	1	1	0	T2N0M0	Moderate diff	Anterior Resection	No				Fistula
2	F	54	5	Middle Rectum	1	2	-1	T3N0M0	Moderate diff	Ultra Low Ant. Resection	ChemoRT/RT	Open cholecystectomy			
3	M	37	3	Middle Rectum	2	3	-1	T2N1M0	Moderate diff	Ultra Low Ant. Resection	ChemoRT/RT			Yes	
4	M	45	4	Lower rectum	1	3	-2	T3N1M0	Moderate diff	Low Anterior Resection	ChemoRT/RT		Cardiac Comorbidities HT	yes	
5	F	50	5	Middle Rectum	1	1	0	T2N0M0	Moderate diff	Anterior Resection	No	Exp Lapr Perf Appendix	Cardiac Comorbidities HT		
6	M	38	3	Lower rectum	0	3	-3	T3N2bM0	Moderate diff	Low Anterior resection	ChemoRT/RT				
7	M	75	7	Middle Rectum	1	3	-2	T4N2M0	Poorly diff	Anterior Resection	ChemoRT/RT	Appendicectomy	HT DM		Cardiovascular Events
8	M	69	6	Middle Rectum	0	2	-2	T3N0M0	Moderate diff	Low Anterior Resection	ChemoRT/RT		DM		
9	F	63	6	Middle Rectum	1	2	-1	T3N0M0	Moderate diff	Ultra Low Ant. Resection	ChemoRT/RT	Exp Lapr Perf DU C/S	DM		
10	M	57	5	Lower rectum	1	1	0	T2N0M0	Moderate diff	Low Anterior Resection	ChemoRT/RT				
11	M	57	5	Middle Rectum	1	3	-2	T3N2M0	Moderate diff	Ultra Low Ant. Resection	ChemoRT/RT				
12	F	63	6	Middle Rectum	0	2	-2	T4aN0M0	Poorly diff	Anterior Resection	ChemoRT/RT		HT		
13	M	28	2	Middle Rectum	2	3	-1	T3N2M0	Moderate diff	Anterior Resection	ChemoRT/RT				
14	F	62	6	Middle Rectum	1	1	0	T1N0M0	Moderate diff	Low Anterior Resection	No	Lap Cholecystectomy			Chest Infection
15	F	50	5	Lower rectum	0	1	-1	T2N0M0	Moderate diff	Low Anterior Resection	ChemoRT/RT	Lap Chole C/S	Cardiac Comorbidities HT	yes	Wound Infection
16	M	60	6	Lower rectum	2	2	0	T3N0M0	Moderate diff	Low Anterior Resection	ChemoRT/RT	Appendicectomy			
17	M	50	5	Middle Rectum	1	1	0	T2N0M0	Moderate diff	Anterior Resection	No				
18	M	40	4	Lower rectum	3	3	0	T3N1M0	Moderate diff	Ultra Low Ant. Resection	ChemoRT/RT	Hemorrhoidectomy		yes	
19	M	65	6	Middle Rectum	1	1	0	T2N0M0	Moderate diff	Anterior Resection	No		Cardiac Comorbidities HT		Cardiovascular Events
20	F	69	6	Middle Rectum	2	3	-1	T2N1M0	Moderate diff	Anterior Resection	ChemoRT/RT		Cardiac Comorbidities DM Renal Impairment		Fistula
21	M	70	7	Middle Rectum	1	3	-2	T2N1M0	Moderate diff	Anterior Resection	ChemoRT/RT	Appendicectomy	Renal Impairment DM	yes	Anastomotic Leak
22	M	60	6	Middle Rectum	1	3	-2	T3N2M0	Moderate diff	Anterior Resection	ChemoRT/RT	Open Chole	DM	yes	Wound Infection
23	M	66	6	Lower rectum	2	3	-1	T3N1M0	Moderate diff	Low Anterior Resection	ChemoRT/RT			yes	
24	F	59	5	Middle Rectum	1	1	0	T2N0M0	Well Diff	Low Anterior Resection	No				
25	F	45	4	Middle Rectum	1	3	-2	T3N2M0	Moderate diff	low Anterior Resection	ChemoRT/RT				
26	F	58	5	Middle Rectum	0	3	-3	T2N1bM0	Moderate diff	Anterior Resection	ChemoRT/RT				
27	F	55	5	Middle Rectum	1	1	0	T2N0M0	Moderate diff	Anterior Resection	No				
28	M	64	6	Lower rectum	2	3	-1	T3N3M0	Moderate diff	AP- Resection	ChemoRT/RT				
29	F	40	4	Middle Rectum	0	0	0	TisN0M0	High Grade Dysplasia	Anterior Resection	No	Lap Chole C/S Hystrectomy	DM COPD		Wound Infection
30	F	60	6	Lower rectum	0	1	-1	T2N0M0	Moderate diff	low Anterior Resection	ChemoRT/RT				
31	F	44	4	Lower rectum	3	3	0	T3N1M0	Moderate diff	low Anterior Resection	ChemoRT/RT	Exp Lapro Perf DU			
32	F	55	5	Middle Rectum	2	3	-1	T3N2M0	Moderate diff	Anterior Resection	ChemoRT/RT				
33	F	25	2	Lower rectum	2	3	-1	T3N1M1	Moderate diff	Low Anterior Resection	ChemoRT/RT	C/S			
34	M	45	4	Lower rectum	1	3	-2	T4bN1bM0	Moderate diff	Low Anterior Resection	ChemoRT/RT		Cardiac Comorbidities HT	yes	
35	M	70	7	Lower rectum	1	3	-2	T2N1M0	Moderate diff	Low Anterior Resection	ChemoRT/RT	Explor Lapro Perf Terminal Ileum	HT	yes	Wound Infection
36	F	55	5	Middle Rectum	1	1	0	T2N0M0	Moderate diff	Anterior Resection	No		HT DM	yes	
37	M	62	6	Middle Rectum	0	3	-3	T2N2aM0	Moderate diff	Anterior Resection	ChemoRT/RT				
38	M	74	7	Middle Rectum	2	3	-1	T3N2aM0	Moderate diff	Anterior Resection	ChemoRT/RT			yes	
39	F	47	4	Lower rectum	0	1	-1	T2N0M0	Moderate diff	Low Anterior Resection	ChemoRT/RT				
40	M	24	2	Middle Rectum	3	3	0	T3N2M0	Moderate diff	Anterior Resection	ChemoRT/RT	Appendicectomy		yes	
41	F	73	7	Middle Rectum	1	3	-2	T3N2M0	Moderate diff	Anterior Resection	ChemoRT/RT				
42	F	68	6	Middle Rectum	1	1	0	T2N0M0	Moderate diff	Anterior Resection	No		HT	yes	
43	F	55	5	Lower rectum	2	3	-1	T4N2M0	Moderate diff	AP- Resection	ChemoRT/RT				
44	M	56	5	Lower rectum	2	3	-1	T3N1M0	Moderate diff	AP- Resection	ChemoRT/RT				
45	F	65	6	Lower rectum	0	1	-1	T2N0M0	Moderate diff	AP- Resection	ChemoRT/RT				
46	F	50	5	Lower rectum	1	1	0	T2N0M0	Well Diff	AP- Resection	ChemoRT/RT		Cardiac Comorbidities HT DM		Abscess
47	F	43	4	Lower rectum	2	3	-1	T3N1M0	Moderate diff	AP- Resection	ChemoRT/RT	Hemorrhoidectomy	DM HT		
48	F	53	5	Lower rectum	0	1	-1	T2N0M0	Moderate diff	AP- Resection	ChemoRT/RT		DM		
49	M	61	6	Lower rectum	2	3	-1	T3N1M0	Moderate diff	AP- Resection	ChemoRT/RT	Appendicectomy	DM HT COPD	yes	
50	M	65	6	Lower rectum	1	3	-2	T2N2aM0	Moderate diff	AP- Resection	ChemoRT/RT				
51	F	63	6	Lower rectum	2	3	-1	T2N1M0	Moderate diff	AP- Resection	ChemoRT/RT			yes	
52	M	47	4	Lower rectum	0	3	-3	T3N2M0	Moderate diff	AP- Resection	ChemoRT/RT				
53	F	40	4	Lower rectum	2	3	-1	T4N2Mo	Moderate diff	AP- Resection	ChemoRT/RT		HT		Wound Infection
54	F	31	3	Lower rectum	3	3	0	T2N2M1	Moderate diff	AP- Resection	ChemoRT/RT	Appendicectomy		Yes	
55	F	65	6	Lower rectum	1	3	-2	T3N1M0	Moderate diff	AP- Resection	ChemoRT/RT		Cardiac Comorbidites HT		Ileus
56	F	54	5	Lower rectum	2	3	-1	T3N2M0	Moderate diff	AP- Resection	ChemoRT/RT		HT DM		
57	F	33	3	Lower rectum	1	3	-2	T3N2bM0	Poorly diff	AP- Resection	ChemoRT/RT				Wound Infection
58	F	66	6	Lower rectum	1	3	-2	T3N2bM1a	Moderate diff	AP- Resection	ChemoRT/RT				
59	M	50	5	Lower rectum	1	3	-2	T3N2bM0	Moderate diff	AP- Resection	ChemoRT/RT		DM Renal Impairment	yes	
60	M	77	7	Lower rectum	1	3	-2	T3N2M0	Moderate diff	AP- Resection	ChemoRT/RT				
61	M	50	5	Lower rectum	2	2	0	T3N0M0	Well Diff	AP- Resection	ChemoRT/RT			yes	Wound Infection
62	M	46	4	Lower rectum	0	0	0	TisN0M0	Moderate diff	AP- Resection	ChemoRT/RT			yes	
63	F	67	6	Lower rectum	0	1	-1	T1NoM0	Moderate diff	AP- Resection	ChemoRT/RT	C/S	Cardiac Comorbidites HT		Cardiovascular Events
64	F	50	5	Lower rectum	1	2	-1	T3N0M0	Moderate diff	AP- Resection	ChemoRT/RT	C/S			
65	M	53	5	Lower rectum	1	3	-2	T3N1M1	Moderate diff	AP- Resection	ChemoRT/RT			yes	
66	M	28	2	Lower rectum	3	3	0	T4N1M0	Poorly diff	AP- Resection	ChemoRT/RT				
67	M	55	5	Lower rectum	1	3	-2	T3N2M0	Moderate diff	AP- Resection	ChemoRT/RT				
68	F	70	7	Middle Rectum	2	3	-1	T3N1M0	Moderate diff	Anterior Resection	ChemoRT/RT				Wound Infection
69	F	50	5	Middle Rectum	1	1	0	T2N0M0	Moderate diff	Anterior Resection	No	Lap Chole C/S, Hystrectomy			
70	M	68	6	Middle Rectum	2	3	-1	T2N1M0	Moderate diff	Ultra Low Ant. Resection	ChemoRT/RT				
71	M	81	8	Middle Rectum	1	3	-2	T4N1M0	Moderate diff	Anterior Resection	ChemoRT/RT		HT		Ileus
72	M	44	4	Middle Rectum	1	1	0	T2N0M0	Moderate diff	Anterior Resection	No				
73	M	58	5	Middle Rectum	2	3	-1	T3N2M0	Moderate diff	Anterior Resection	ChemoRT/RT				
74	M	64	6	Middle Rectum	0	3	-3	T2N1aM0	Moderate diff	Anterior Resection	ChemoRT/RT		DM	yes	
75	M	69	6	Middle Rectum	2	3	-1	T1N1M0	Moderate diff	Anterior Resection	ChemoRT/RT				
76	F	51	5	Middle Rectum	1	3	-2	T2bN1M0	Poorly diff	Anterior Resection	ChemoRT/RT				
77	M	73	7	Middle Rectum	1	1	0	T2N0M0	Moderate diff	Ultra Low Ant. Resection	No		Cardiac Comorbidities HT DM		Chest Infection
78	M	46	4	Middle Rectum	1	3	-2	T4aN2bM0	Moderate diff	Anterior Resection	ChemoRT/RT				
79	M	62	6	Middle Rectum	1	2	-1	T4N0M0	Moderate diff	Anterior Resection	ChemoRT/RT		Cardiac Comorbidites HT DM	Yes	Mortality
80	F	55	5	Middle Rectum	1	3	-2	T2N1M0	Moderate diff	Anterior Resection	ChemoRT/RT				
81	M	43	4	Middle Rectum	2	3	-1	T1N1M0	Moderate diff	Anterior Resection	ChemoRT/RT				
82	F	56	5	Middle Rectum	1	1	0	T2NoM0	Moderate diff	Anterior Resection	No	C/S	Cardiac Comorbidities HT		
83	M	32	3	Middle Rectum	1	3	-2	T1N1M0	Moderate diff	Anterior Resection	ChemoRT/RT		DM		
84	M	60	6	Middle Rectum	3	3	0	T3N2M0	Moderate diff	Anterior Resection	ChemoRT/RT		DM HT	yes	
85	M	26	2	Middle Rectum	1	1	0	T2N0M0	Well Diff	Anterior Resection	No			yes	
86	F	32	3	Middle Rectum	1	3	-2	T3N1M0	Moderate diff	Anterior Resection	ChemoRT/RT				
87	F	67	6	Lower rectum	0	2	-2	T1N0M0	Moderate diff	Low Anterior Resection	ChemoRT/RT			yes	
88	M	50	5	Lower rectum	2	3	-1	T2N1M0	Moderate diff	low Anterior Resection	ChemoRT/RT			yes	
89	F	54	5	Middle Rectum	1	2	-1	T3N0M0	Moderate diff	Anterior Resection	ChemoRT/RT		DM		

## References

[1] Araghi M, Soerjomataram I, Jenkins M, Brierley J, Morris E, Bray F, Arnold M (2019). Global trends in colorectal cancer mortality: projections to the year 2035. Int J Cancer.

[2] (2014). Cancer in Australia 2021. https://www.aihw.gov.au/reports/cancer/cancer-in-australia-an-overview-2014/summary.

[3] Yang X, Zhang G, Jiang L (2018). Laparoscopic sphincter-saving surgery for low rectal cancer through marker meeting approach. Ann Transl Med.

[4] Perry WB, Connaughton JC (2007). Abdominoperineal resection: how is it done and what are the results?. Clin Colon Rectal Surg.

[5] Heald RJ, Husband EM, Ryall RD (1982). The mesorectum in rectal cancer surgery--the clue to pelvic recurrence?. Br J Surg.

[6] Pilipshen SJ, Heilweil M, Quan SH, Sternberg SS, Enker WE (1984). Patterns of pelvic recurrence following definitive resections of rectal cancer. Cancer.

[7] Delibegovic S (2017). Introduction to total mesorectal excision. Med Arch.

[8] Shin US, Kim CW, Yu CS, Kim JC (2010). Delayed anastomotic leakage following sphincter-preserving surgery for rectal cancer. Int J Colorectal Dis.

[9] Yu JH, Huang XW, Song YC, Lin HZ, Zheng FW (2022). Analysis of prevention and treatment of anastomotic leakage after sphincter-preserving surgery for middle- and low-grade rectal cancer under laparoscopy. Int J Clin Pract.

[10] Wang J, Long Y, Liu K, Pei Q, Zhu H (2021). Comparing neoadjuvant long-course chemoradiotherapy with short-course radiotherapy in rectal cancer. BMC Gastroenterol.

[11] Kim MJ, Lee DW, Kang HC (2023). Total neoadjuvant therapy with short-course radiotherapy versus long-course neoadjuvant chemoradiotherapy in locally advanced rectal cancer, Korean trial (TV-LARK trial): study protocol of a multicentre randomized controlled trial. BMC Cancer.

[12] Purim O, Gordon N, Brenner B (2013). Cancer of the colon and rectum: potential effects of sex-age interactions on incidence and outcome. Med Sci Monit.

[13] Kleemann M, Benecke C, Helfrich D, Bruch HP, Keck T, Laubert T (2014). Prospective analysis of more than 1,000 patients with rectal carcinoma: are there gender-related differences?. Viszeralmedizin.

[14] Bokey EL, Chapuis PH, Fung C, Hughes WJ, Koorey SG, Brewer D, Newland RC (1995). Postoperative morbidity and mortality following resection of the colon and rectum for cancer. Dis Colon Rectum.

[15] Alves A, Panis Y, Mathieu P, Kwiatkowski F, Slim K, Mantion G (2005). Mortality and morbidity after surgery of mid and low rectal cancer: results of a French prospective multicentric study. Gastroenterologie clinique et biologique.

[16] Paun BC, Cassie S, MacLean AR, Dixon E, Buie WD (2010). Postoperative complications following surgery for rectal cancer. Ann Surg.

[17] Tsujimoto H, Ueno H, Hashiguchi Y, Ono S, Ichikura T, Hase K (2010). Postoperative infections are associated with adverse outcome after resection with curative intent for colorectal cancer. Oncol Lett.

[18] Serra-Aracil X, García-Domingo MI, Parés D (2011). Surgical site infection in elective operations for colorectal cancer after the application of preventive measures. Arch Surg.

[19] Vermeer TA, Orsini RG, Daams F, Nieuwenhuijzen GA, Rutten HJ (2014). Anastomotic leakage and presacral abscess formation after locally advanced rectal cancer surgery: incidence, risk factors and treatment. Eur J Surg Oncol.

[20] Muratore A, Mellano A, Marsanic P, De Simone M (2015). Transanal total mesorectal excision (taTME) for cancer located in the lower rectum: short- and mid-term results. Eur J Surg Oncol.

